# The Tariff on Dental Goods

**Published:** 1890-12

**Authors:** 


### The Tariff on Dental Goods.

The increased duty put upon dental goods, and especially upon artificial teeth by the recently enacted tariff law, is eliciting considerable criticism, by dentists, especially by those who have found it desirable to use some imported goods. There has been as it would seem, in reasoned justice, a sufficient duty heretofore on this class of imports, viz: from 20 per cent. to 45 per cent. ad valorum. This ought to afford sufficient protection to American Dental Manufacturers; and especially does it seem so, when we consider that immense quantities of dental goods of all sorts are sold in European countries, practically free of duty. But it would seem that the former tariff was not sufficient in amount and it has been increased three-fole which will have the effect of excluding, nearly, if not quite all such goods from the profession of the United States. All surgical and dental instruments and appliances used in the irritigation and relief of human suffering, should have free passage so far as tariff is concerned, into and through all civilized countries of the world.

